# Abscess in Adenomyosis Mimicking a Malignancy in a 54-Year-Old Woman

**DOI:** 10.1155/S1064744903000085

**Published:** 2003

**Authors:** Rezzan Erguvan, Mehmet M. Meydanli, Alpay Alkan, Mehmet N. Edali, Hasan Gokce, Ayşe Kafkasli

**Affiliations:** 1 Department of Pathology School of Medicine Inonu University Turkey; 2 Department of Obstetrics and Gynecology School of Medicine Inonu University Turkey; 3 Department of Radiology School of Medicine Inonu University Malatya Turkey; 4 Cosnuk Mah Mehmet Buyruk Cad Resat Turgut Sitesi B-Blok No:75 D:26 Malatya TR-44330 Turkey

## Abstract

*Background*: Although there are a few reports describing abscess formation in endometriotic foci no report of abscess formation arising *de novo* within adenomyosis appears in the literature. Preoperative diagnosis of adenomyosis is frequently difficult because of non-specific signs and symptoms. Synchronous pelvic pathologies such as leiomyoma, endometrial polyp, endometrial hyperplasia, as well as endometrial cancer may cause differential diagnostic problems.

* Case*: A 54-year-old postmenopausal woman complaining of inguinal pain, nightsweats and hot flashes is presented. Radiologic examinations of the pelvis revealed a 95 × 85 mm leiomyoma-like lesion including a 53 × 43 mmcystic space and 9 × 6 mmpapillary formation within the uterus raising clinical suspicion of malignancy. A total abdominal hysterectomy and bilateral salpingo-oophorectomy were performed accompanied by a frozen section diagnosis. The frozen section revealed an abscess formation arising in a focus of adenomyosis. The postoperative period of the patient was uneventful.

*Conclusion* : The present case, to our knowledge, is the first report representing abscess formation in adenomyosis. Abscess arising within adenomyosis can strongly raise the suspicion of endometrial cancer, particularly if the patient is postmenopausal. If endometrial cancer cannot be ruled out with definitive histopathological diagnosis in the preoperative period, a frozen section becomes mandatory during surgical intervention.


*Infect Dis Obstet Gynecol 2003;11:59–64*

### Abscess in adenomyosis mimicking a malignancy in a 54-year-old woman

**Rezzan Erguvan[1], Mehmet M. Meydanli[2], Alpay Alkan[3], Mehmet N. Edali[1], Hasan Gokce[1] and Ayşe Kafkasli[2]**

[1]*Department of Pathology*
[2]*Department of Obstetrics and Gynecology and*
[3]*Department of Radiology, School of Medicine, Inonu University, Malatya, Turkey*

***Background:*** Although there are a few reports describing abscess formation in endometriotic foci no report of abscess formation arising *de novo* within adenomyosis appears in the literature. Preoperative diagnosis of adenomyosis is frequently difficult because of non-specific signs and symptoms. Synchronous pelvic pathologies such as leiomyoma, endometrial polyp, endometrial hyperplasia, as well as endometrial cancer may cause differential diagnostic problems.
***Case:*** A 54-year-old postmenopausal woman complaining of inguinal pain, nightsweats and hot flashes is presented. Radiologic examinations of the pelvis revealed a $95 \times 85$ mm leiomyoma-like lesion including a $53 \times 43$ mm cystic space and $9 \times 6$ mm papillary formation within the uterus raising clinical suspicion of malignancy. A total abdominal hysterectomy and bilateral salpingo-oophorectomy were performed accompanied by a frozen section diagnosis. The frozen section revealed an abscess formation arising in a focus of adenomyosis. The postoperative period of the patient was uneventful.
***Conclusion:*** The present case, to our knowledge, is the first report representing abscess formation in adenomyosis. Abscess arising within adenomyosis can strongly raise the suspicion of endometrial cancer, particularly if the patient is postmenopausal. If endometrial cancer cannot be ruled out with definitive histopathological diagnosis in the preoperative period, a frozen section becomes mandatory during surgical intervention.

Key words: FROZEN SECTION; TRANSVAGINAL ULTRASONOGRAPHY; UTERUS; SURGERY

### INTRODUCTION

Adenomyosis is a common gynecological condition that is characterized by the presence of endometrial glands and stroma extending beneath the endomyometrial interface which nests deep within the myometrium, whereas endometriosis refers to the occurrence of endometrial tissue outside the uterus[1–5] or on the uterine serosa[4]. Despite its high frequency, etiology of adenomyosis is still unknown. The current leading theory suggests adenomyosis develops as a result of down-growth and invagination of the basalis endometrium into the myometrium[6].

Preoperative diagnosis of adenomyosis is frequently difficult because of non-specific signs and symptoms. Synchronous pelvic pathologies such as leiomyoma, endometrial polyp, endometrial hyperplasia as well as endometrial cancer may

Rezzan Erguvan, MD, Cosnuk Mah, Mehmet Buyruk Cad, Resat Turgut Sitesi B-Blok, No:75 D:26, TR-44330, Malatya, Turkey. Email: rerguvan@yahoo.com

© 2003 The Parthenon Publishing Group 

cause differential diagnostic problems. This difficulty arises especially if the patient is postmenopausal with established risk factors for endometrial cancer. The respective diagnosis is rendered more difficult in the case of inadequate biopsy specimen for the definitive diagnosis while the radiologic findings favor a primary uterine cancer.

Although there are a few reports describing abscess formation in endometriotic foci[3,7,8], no report of abscess formation arising *de novo* within adenomyosis appears in the literature. In our report a 54-year-old woman with a suspected malignancy was discovered as having adenomyosis and abscess formation arising *de novo* within. This is the first case in the literature showing adenomyosis including abscess formation.

### CASE REPORT

A 54-year-old, multiparous (gravida 4, para 3, induced abortus 1), obese, postmenopausal woman complaining of inguinal pain, nightsweats and hot flashes was admitted to Inonu University Gynecology and Obstetrics Clinic in November 2001. Chronic obstructive lung disease, diabetes mellitus and hypertension were present in the history whereas no history of hormone replacement therapy was noted. No history of pelvic inflammatory disease, intrauterine device, endometriosis, tubal pathology or diverticular disease was noted.

The duration of menopause was defined to be 14 months with the patient's menopause age being > 52 years. Except for the presence of an irregularly enlarged uterus, normal findings were detected when a gynecological examination was performed.

Transvaginal ultrasonography revealed a 95 × 85 mm leiomyoma-like lesion including a 53 × 43 mm cystic space (Figure 1a) and 9 × 6 mm papillary formation within the uterus (Figure 1b). Because of distortion of the endometrial cavity by the lesion described, endometrial thickness could not be assessed. An endometrial biopsy was performed and the pathologic examination revealed inadequate tissue for the definitive diagnosis. The patient was hospitalized in order to rule out a uterine malignancy.

Laboratory results including coagulation panel and biochemical tests did not reveal any data of

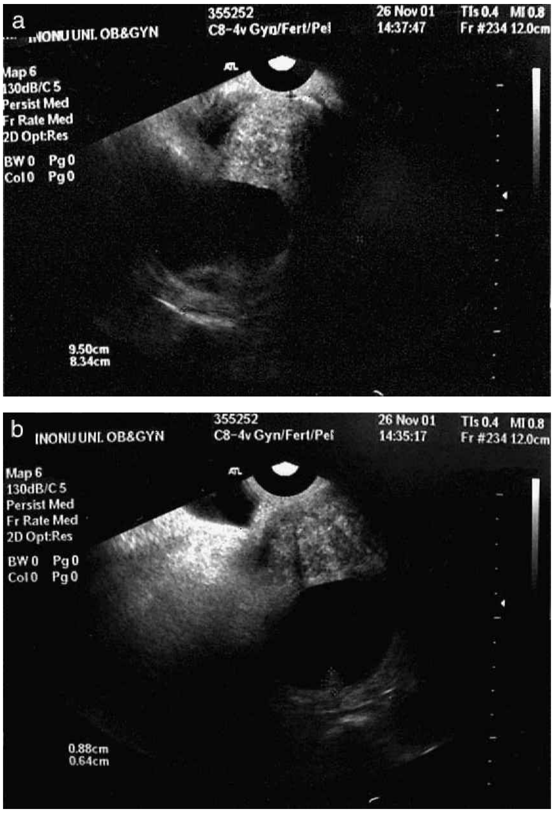

**Figure 1** Transvaginal ultrasonography revealed a 95 × 85 mm leiomyoma-like lesion consisting of a 54 × 43 mm cystic space (a), in which a 9 × 6 mm papillary structure (b) was noted interest except for the presence of hyperglycemia. The leukocyte count was 7600/mm$^3$ with neutrophils 68.8%, lymphocytes 26.7%, monocytes 3%, and eosinophils 1.5%. The patient was not febrile during her workup. Mammography and other laboratory examinations showed no abnormal signs. Tumor markers including CA 125 were reported to be within normal ranges.

Magnetic resonance imaging of the pelvis was performed subsequently and the uterus was detected as greater than normal, having lobulated contours and heterogen density. In addition to intramural lesions with the greatest diameter of 5 cm, cystic and necrotic degenerations consistent with leiomyoma that obstruct the endocervical canal and a diffuse fluid accumulation in the endometrial cavity were noted. Ovaries were normal bilaterally.

A 5 × 5 mm polypoid lesion on the anterior and posterior wall of the uterine fundus was detected upon hysterosonography. Additionally a 1 × 1 cm anechoic structure protruding into the endometrial cavity in fundus was detected.

A fractional dilatation and curettage was performed under general anesthesia but the pathologic examination revealed inadequate tissue. A hysteroscopy was not performed because of the obstruction of the endocervical canal by one of the lesions described. Since it was not possible to exclude uterine malignancy with definitive histopathological diagnosis, the patient underwent exploratory laparotomy for clinically assumed uterine malignancy. A total abdominal hysterectomy and bilateral salpingo-oophorectomy were performed accompanied by a frozen section diagnosis. The patient had no fever during the surgery. Intraoperative findings did not suggest any evidence of pelvic tuberculosis, nor was systemic tuberculosis detected. The frozen section revealed an abscess formation arising in a focus of adenomyosis. The postoperative period of the patient was uneventful.

### METHODS

### Diagnostic evaluation

The material was fixed in 10% buffered formalin and processed routinely. Hematoxylin-eosin stained slides were examined.

Macroscopically 238 g weight and 23 × 10 × 5.5 cm specimen was evaluated. Thickness of endometrium was 1 mm and it was 2 cm for myometrium. Serial sections revealed a 2 cm cystic lesion localized in myometrium adjacent to endometrium in the vicinity of left cornu uteri (Figure 2). A purulent material was drained during the section. The wall of the cyst had a greyish white–yellow color and an irregular appearance. No papillary formation on the wall was identified. The right ovary had a 1 cm cystic formation. In addition a 7 mm right paratubal cyst was noted. The left ovary and fallopian tube were grossly unremarkable.

Histologically beneath the basal layer of the endometrium there were foci of endometrial glands embedded in their own stroma extending deep within the myometrium. The lumina of these glands contained polymorphonuclear leukocytes infiltrating the glandular epithelium and the stroma (Figure 3). The cavity was truly an abscess cavity, but not necrotic debris from a degenerating

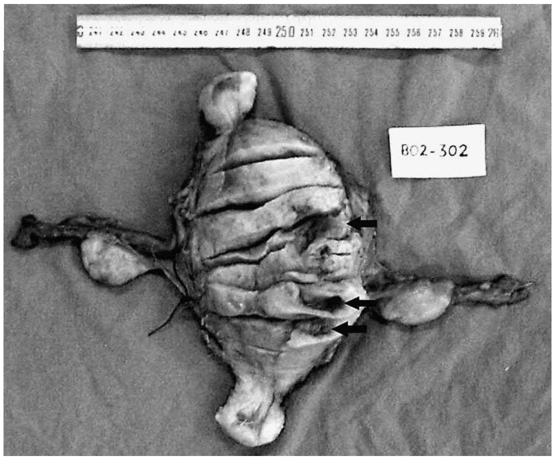

**Figure 2** The uterus is seen interiorly revealing a 2 cm cystic lesion localized in the myometrium adjacent to the endometrium in the vicinity of left cornu uteri. Arrows show the cystic cavity in serial sections

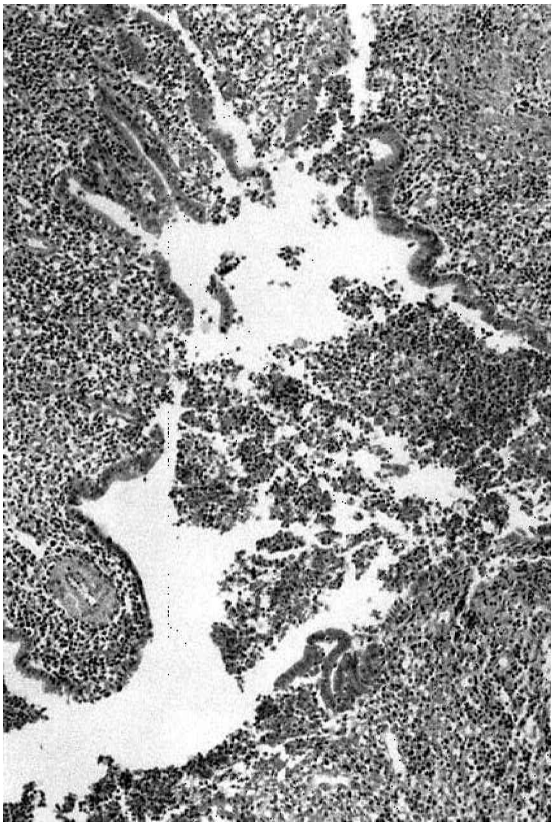

**Figure 3** Abscess formation in adenomyosis (hematoxylin-eosin × 100)

leiomyoma. A tissue section obtained under sterile conditions was cultured for both aerobic and anaerobic microorganisms, but results were inconclusive. The final diagnosis was abscess formation in an adenomyosis focus confirming the results of the frozen section in this case.

### DISCUSSION

Adenomyosis is a condition in which endometrial glands and stroma are located inordinately deep in the myometrium. Endometriosis is defined as endometrial tissue occurring ectopically outside the uterus[1–5,9] or on the uterine serosa[4].

It is generally accepted that about 15% of multiparous women develop varying degrees of adenomyosis in their late 30s and early 40s[1,3,5,10]. About 15% of women with adenomyosis have associated endometriosis[10]. Previous studies have shown that the mean age in endometriosis is between 35–39 years and women are frequently multiparous[5,11]. Our patient is different in that she has presented with adenomyosis postmenopausally at 54 years of age.

Although adenomyosis and endometriosis are regarded as closely related, their microscopic appearance and pathogenesis are somewhat different. It is known that they often occur independently of each other. While adenomyosis is made up of the non-functional (basal) layer of the endometrium, endometriosis is composed of the functional layers so that proliferative, secretory and menstrual changes can be seen in the latter[2].

Since in some cases of adenomyosis, the affected endometrium is functional, the most important consequence of adenomyosis is shedding of the endometrium during the menstrual cycle[1,12]. As a result of hemorrhage within the adenomyotic foci, menorrhagia, colicky dismenorrhea, dispareunia and pelvic pain occur especially during the premenstrual period[1,4,5,9]. Egger and Weigmann[11] state that the pain during menstruation is due to uterine dyskinesia. However, about 30–40% of patients with adenomyosis are asymptomatic; hence, this disease can be a surprise pathologic finding in a patient without menorrhagia, dysmenorrhea, or uterine enlargement[10]. The pain in the present case was not of a pelvic origin, but an inguinal pain. Since there were no other findings to cause inguinal pain, this pain may have been caused by the abscess itself. Foci of adenomyosis may be affected by other diseases affecting the orthotopic endometrium (e.g., hyperplasia or malignant changes). Therefore, it is important not to misinterpret such a condition as a deeply invasive malignancy[2,4,5]. These foci can also become decidualized during pregnancy[5]. Adenomyosis in pregnancy can lead to rupture of membranes[2].

When the uterus is diffusely involved by adenomyosis, it is enlarged in a globoid fashion[4,5,9]. The most commonly affected site is the posterior wall[5,9]. If it is focal ('adenomyoma'), it mimics a leiomyoma showing a roughly spherical, intramural, space-occupying lesion. Its difference from leiomyoma is not being shelled out easily from the surrounding uninvolved myometrium[4,5].

There are rare cases reported in the literature describing the presence of abscess in endometriosis in different localizations, most of them being in the ovaries[3,7,8] but none in adenomyosis, to our knowledge.

Martino and colleagues[7] reported a 16-year-old girl who underwent surgery because of congenital anomalies of the bladder, rectum and vagina. In the follow-up a right lower quadrant mass was discovered and subsequently they detected three cystic midline masses at the level of umbilicus. Following the examination procedures including fine-needle aspiration (FNA) biopsies of these lesions, they detected a secondary infection of endometriosis arising from the fallopian tube and the ovary. Laboratory results revealed that the etiologic agent was clostridium. They interpreted that it was an iatrogenic infection following FNA biopsy[7].

Lipscomb and associates[8] described an abscess measuring as large as 20 cm in the ovary and they emphasized that it was the first case in the literature arising *de novo* within an endometriosis in their report. They also stated that it was the second case in the literature leading to ureteral obstruction. They hypothesized that the abscess in their case might have developed following hematogenous spread of bacteria from a urinary tract infection because the same organism was recovered from both the patient's urine and the abscess cavity[8].

Egger and Weigmann[11] found that the incidence of infection in endometriotic cysts, formation of an isolated ovarian abscess, was ranging between 8–18% in their study of 263 patients. They stated that earlier surgical treatment of endometriotic cysts might lead to decrease in the risk of secondary infection[11].

There are some other reports describing perforation of the colon due to endometriosis[3,13]. Floberg and co-workers[13] reported a 34-year-old woman who had an uneventful delivery and developed perforation of the colon immediately in the postpartum stage. They detected a 5 cm abscess causing enlargement of the left ovary which was adhering to the sigmoid colon in the laparotomy performed. They stated that the abscess had ruptured into the peritoneal cavity and there was also a fistula from the abscess to the colon. They discovered that the microorganisms that led to infection were Gram-negative bacteria. Since they did not find any other focus of infection like tuba uterina which might transmit to the ovary, they assumed that the ovary became infected from the colon via the fistula. They reported that the patient recovered after receiving broad-spectrum antibiotics intravenously[13].

In our patient, because there were no microorganisms isolated from the foci of abscess in both aerobic and anaerobic cultures, and there was no other source to lead a secondary suppuration including particularly the uterus itself, the cause of infection remained unknown. But it is clear in this case that this was a primary abscess formation in the adenomyosis defined for the first time.

Treatment modality for adenomyosis is primarily surgical[5]. Because abscess formation was localized within the adenomyotic foci, it was regarded that antibiotic therapy was unnecessary, and total abdominal hysterectomy and bilateral salpingo-oophorectomy was performed. Thus the patient ameliorated after surgery and was discharged without any complications.

In conclusion, abscess arising within adenomyosis can strongly raise the suspicion of endometrial cancer, particularly if the patient is postmenopausal. If endometrial cancer cannot be ruled out with definitive histopathological diagnosis in the preoperative period, a frozen section becomes mandatory during surgical intervention.

### REFERENCES

1. Crum CP. The female genital tract. In Cotran RS, Kumar V, Collins T, eds. *Robbins Pathologic Basis of Disease*, 6th edn. Philadelphia, Pennsylvania: W.B. Saunders Co., 1999:1035–91
2. Rosai J. Female reproductive system. In Rosai J, ed. *Ackerman's Surgical Pathology*, 8th edn. Missouri: Mosby-Year Book Inc., 1996:1319–564
3. Ledley GS, Shenk IM, Heiy HA. Sigmoid colon perforation due to endometriosis not associated with pergnancy. *Am J Gastroenterol* 1988;83: 1424–6
4. Hendrickson MR, Longacre TA, Kempson RL. The uterine corpus. In Sternberg SS, ed. *Diagnostic Surgical Pathology*, 3rd edn. Philadelphia: Lippincott Williams & Wilkins, 1999:2203–5
5. Hendrickson MR, Kempson RL. Non-neoplastic conditions of the myometrium and uterine serosa. In Fox H, ed. *Obstetrical and Gynaecological Pathology*, 4th edn. Churchill-Livingstone, 1995:511–18
6. Ferenczy A. Pathophysiology of adenomyosis. *Hum Reprod* 1998;4:312–22
7. Martino CR, Haaga JR, Bryan PJ. Secondary infection of an endometrioma following fine-needle aspiration. *Radiology* 1984;151:53–4
8. Lipscomb GH, Ling FW, Photopulos GJ. Ovarian abscess arising within an endometrioma. *Obstet Gynecol* 1991;78:951–4
9. Kraus FT. Female genitalia. In Kissane JM, ed. *Anderson's Pathology*, 8th edn. Mosby, 1985: 1451–545
10. Moore JG. Endometriosis and adenomyosis. In Hacker NF, Moore JG, eds. *Essentials of Obstetrics and Gynecology*, 3rd edn. Philadelphia: WB Saunders Co., 1998:432–40

11. Egger H, Weigmann P. Clinical and surgical aspects of ovarian endometriotic cysts. *Arch Gynecol* 1982;233:37–45
12. Cotran RS, Kumar V, Robbins SL. Female genital tract. In Cotran RS, Kumar V, Robbins SL, eds. *Robbins–Pathologic Basis of Disease*, 4th edn. Philadelphia: W.B. Saunders Co., 1989:1127–80
13. Floberg J, Backdahl M, Silfersward C, Thomassen PA. Postpartum perforation of the colon due to endometriosis. *Acta Obstet Gynecol Scand* 1984; 63:183–4

RECEIVED 07/08/02; ACCEPTED 02/01/03
